# Fundus Image Deep Learning Study to Explore the Association of Retinal Morphology with Age-Related Macular Degeneration Polygenic Risk Score

**DOI:** 10.3390/biomedicines12092092

**Published:** 2024-09-13

**Authors:** Adam Sendecki, Daniel Ledwoń, Aleksandra Tuszy, Julia Nycz, Anna Wąsowska, Anna Boguszewska-Chachulska, Andrzej W. Mitas, Edward Wylęgała, Sławomir Teper

**Affiliations:** 1 Chair and Clinical Department of Ophthalmology, Faculty of Medical Sciences in Zabrze, Medical University of Silesia, 40-752 Katowice, Poland; adam.sendecki@gmail.com (A.S.); ewylegala@sum.edu.pl (E.W.); slawomir.teper@sum.edu.pl (S.T.); 2 Faculty of Biomedical Engineering, Silesian University of Technology, 41-800 Zabrze, Poland; aleksandra.tuszy@polsl.pl (A.T.); andrzej.mitas@polsl.pl (A.W.M.); 3 Institute of Biomedical Engineering and Informatics, Technische Universität Ilmenau, 98693 Ilmenau, Germany; julia.nycz@tu-ilmenau.de; 4 Department of Bioinformatics, Polish-Japanese Academy of Information Technology, 02-008 Warszawa, Poland; 5 Genomed S.A., 02-971 Warszawa, Poland; 6 Department of Scientific Research, Branch in Bielsko-Biala, Medical University of Silesia, 40-752 Katowice, Poland

**Keywords:** artificial intelligence, age-related macular degeneration, deep learning, polygenic risk score, retinal imaging, fundus images

## Abstract

Background: Age-related macular degeneration (AMD) is a complex eye disorder with an environmental and genetic origin, affecting millions worldwide. The study aims to explore the association between retinal morphology and the polygenic risk score (PRS) for AMD using fundus images and deep learning techniques. Methods: The study used and pre-processed 23,654 fundus images from 332 subjects (235 patients with AMD and 97 controls), ultimately selecting 558 high-quality images for analysis. The fine-tuned DenseNet121 deep learning model was employed to estimate PRS from single fundus images. After training, deep features were extracted, fused, and used in machine learning regression models to estimate PRS for each subject. The Grad-CAM technique was applied to examine the relationship between areas of increased model activity and the retina’s morphological features specific to AMD. Results: Using the hybrid approach improved the results obtained by DenseNet121 in 5-fold cross-validation. The final evaluation metrics for all predictions from the best model from each fold are MAE = 0.74, MSE = 0.85, RMSE = 0.92, R^2^ = 0.18, MAPE = 2.41. Grad-CAM heatmap evaluation showed that the model decisions rely on lesion area, focusing mostly on the presence of drusen. The proposed approach was also shown to be sensitive to artifacts present in the image. Conclusions: The findings indicate an association between fundus images and AMD PRS, suggesting that deep learning models may effectively estimate genetic risk for AMD from retinal images, potentially aiding in early detection and personalized treatment strategies.

## 1. Introduction

Age-related macular degeneration (AMD) is the leading cause of vision loss in people over 50 in developed countries, affecting around 67 million people in the European Union [1,2,3,4,5]. Due to population aging, this number is expected to increase by 15% in the coming years [6]. By 2040, it is estimated that 288 million people globally will be affected by AMD [7,8]. Currently, 25.3% of people over 60 in Europe have early or intermediate AMD, while 2.4% have late AMD. Among those aged 70 and older, 13.2% have early AMD, and 3.0% have late AMD [7,8]. At the same time, in Poland, approximately 0.65% of the population over 65 requires treatment with VEGF inhibitors for AMD with macular neovascularization [9].

Depending on the severity of the disease, early, intermediate, and late AMD are distinguished. Late forms of AMD can be either neovascular (nAMD) or non-neovascular (non-nAMD). Neovascular AMD is less common, accounting for only 10–15% of all cases, and it is the only form that can be treated pharmacologically [10]. Due to the progression of the disease, early screening and detection of those at risk are essential to prevent vision loss. However, the screening of AMD is limited by a shortage of human assessors, limited coverage of screening programs, and financial constraints [11].

Diagnosing AMD involves a combination of methods to identify signs of the disease, including imaging techniques such as optical coherence tomography (OCT), fluorescein angiography, OCT-angiography, and color fundus photography. Based on this, much research is being conducted in computer-assisted AMD diagnosis using image processing methods and artificial intelligence [12]. Proposed approaches include machine learning techniques based on quantitative features that characterize retinal morphology in various aspects. Often, feature extraction is preceded by segmenting individual retinal structures and layers on images using deep learning models. Another approach uses deep learning directly on the images to account for the many different morphological factors affecting the onset and progression of AMD [13]. Concerning fundus images, the proposed deep learning techniques have high performance in lesion segmentation and classification of AMD and its severity [14,15]. Moreover, a study by Zekavat et al. proved that deep learning can quantify fundus images for integration with genetic data to inform disease risk prediction and modification [16].

Research has shown that genetic variations influence an individual’s susceptibility to AMD. Specific genes linked to biological processes such as inflammation, lipid metabolism, and the complement system have been identified as associated with the disease. Variations in these genes can affect the function and regulation of important pathways that maintain retinal health. Additionally, studies suggest that AMD tends to cluster within families, indicating a strong genetic component. As a result, close relatives of individuals with AMD are at a higher risk of developing the disease than the general population [17].

Over the years, numerous genes have been identified as playing a role in the pathogenesis of AMD, including complement factor H (CFH), age-related maculopathy susceptibility 2 (ARMS2), high-temperature requirement A-1 (HTRA1), and complement component 3 (C3), among others [18,19,20]. In addition, a large-scale meta-analysis by He et al. uncovered new loci associated with AMD, further advancing our understanding of the genetic basis of the disease [21].

One method for stratifying genetic predisposition to a disease is the calculation of the polygenic risk score (PRS), which integrates the weighted impact of multiple genetic variants. It combines the small or moderate effects of numerous genetic variants into a single score that predicts an individual’s risk relative to the population. The PRS is estimated based on the effect sizes of risk alleles derived from genome-wide association studies (GWAS) summary statistics of related traits with shared genetic etiology, along with genotyping data from a target group of patients, which may include whole genome, exome, or targeted sequencing or array data [21,22]. By aggregating the effect sizes of multiple risk-associated single nucleotide polymorphisms, the PRS estimates an individual’s genetic predisposition to a particular condition, such as AMD. GWAS have significantly contributed to our understanding of the genetic architecture of AMD by identifying and validating numerous AMD-associated genetic variants, which are then used in the calculation of PRS [23,24,25]. In the case of AMD, the PRS can provide valuable insights into an individual’s genetic susceptibility and risk of developing the disease [21]. Additionally, PRS assessments have been successfully applied in several medical studies, including those focused on cognitive function [26], schizophrenia, breast cancer [27], lung cancer [28], and hemophilic arthropathy [29].

The search for a relationship between morphological features of the retina and AMD PRS is a relatively new area of research. Current studies using features determined from OCT images have shown a relationship between AMD PRS and outer retinal layer thickness [30] and photoreceptor layer thickness [31,32]. Moreover, our previous studies using deep learning also preliminarily confirmed a relationship between deep features from OCT images and AMD PRS, providing a basis for continued work on this issue [33].

This article aims to investigate the relationship between AMD PRS and changes in the central retina seen in fundus images using deep learning techniques. We assume that supporting the study with deep learning makes it possible to observe potential associations that still need to be explored due to the complexity of the factors contributing to AMD. The proposed approach requires developing and verifying a deep-learning model to estimate PRS from fundus images. The use of explainability techniques allows for a comparison of the regions of the retina considered relevant to the estimated PRS value with the actual condition of the retina and the true PRS obtained as a result of the genetic analysis of patients’ blood samples.

## 2. Materials and Methods

### 2.1. Study Population

Participants in the study were recruited from the Chair and Clinical Department of Ophthalmology at the Faculty of Medical Sciences in Zabrze, part of the Medical University of Silesia in Katowice. Ethical approval for the study was granted by the Ethics Committee of the Medical University of Silesia (Resolutions No KNW/0022/KB1/105/13 and BNW/NWN/0052/KB1/97/I/22/23), following the guidelines of the Declaration of Helsinki. Each participant signed a written informed consent form after receiving comprehensive information about the study protocol.

The study group consisted of 332 individuals, with 235 diagnosed with age-related macular degeneration (AMD) and 97 healthy controls who showed no symptoms of retinal degeneration. Among AMD patients, the stages of the disease varied, including early and intermediate, as well as advanced forms, such as neovascular AMD, geographic atrophy, and subretinal fibrosis. The control group consisted of individuals without AMD symptoms, recruited during follow-up visits after cataract surgery or routine check-ups, where fundus examinations confirmed the absence of AMD.

### 2.2. Exclusion Criteria

The study excluded individuals with macular conditions other than AMD, such as diabetic macular edema or macular dystrophy. Additional exclusion criteria included vision impairments that prevented proper fundus evaluation, a history of retinal or choroidal inflammatory diseases, retinal detachment, previous intraocular surgeries aside from cataract surgery or posterior capsulotomy, and previous retinal laser therapy.

### 2.3. Ophthalmological Examinations

The study included a range of ophthalmological assessments, such as best-corrected visual acuity (BCVA) evaluation using ETDRS charts, examination of the anterior segment with slit lamp biomicroscopy, and pupillary dilation with 1% tropicamide. Each eye underwent a thorough clinical examination using Volk superfield aspheric lenses 90D to evaluate the posterior segments and detect any pathological changes in the macula.

### 2.4. Imaging Techniques

Fundus images, swept-source optical coherence tomography (SS-OCT) of the macula (utilizing both radial and 3D wide scanning protocols), and optical coherence tomography angiography (OCTA) were captured using the DRI OCT Triton tomograph (Topcon Healthcare, Tokyo, Japan). A collection of color fundus images covering the optic disc and central retina was also acquired routinely during the OCT and OCTA procedures.

### 2.5. Polygenic Risk Score Calculation

Ulańczyk et al. conducted an extensive study on 30 genes linked to essential retinal functions, including the regulation of inflammation (e.g., TGFB1), immune response (e.g., C2, C3, CFB, CFH), lipid and protein synthesis (e.g., ELOVL4, HTRA1, RPL1), and maintaining the structural integrity of the retinal layer (e.g., BEST1, C1QTNF5, GUCA1B). The research also examined genes involved in oxidative stress, extracellular matrix maintenance, transmembrane transport, transcription regulation, DNA repair, and AMD (e.g., ARMS2). Molecular inverted probes were used to enrich coding regions and their flanking sequences, followed by sequencing on the Illumina platform. The targeted enrichment of coding sequences for these 30 AMD-related genes was performed at Genomed S.A., Warsaw [34].

The bioinformatics analysis included adapter trimming using Cutadapt v1.14 [35], mapping reads to the GRCh37.13 reference genome with the Burrows–Wheeler Aligner v0.7.10, and deduplication using unique molecular identifiers through in-house scripts. Further steps involved indel realignment and base recalibration using the Genome Analysis Toolkit v3.5 (GATK) [36], a widely recognized tool for ensuring best practice and maintaining the highest quality and accuracy of the results.

Variant calling was performed using the HaplotypeCaller and UnifiedGenotyper tools from the GATK package to accurately identify single nucleotide variants and indels. Rigorous criteria were applied, such as excluding variants missing in <95% of samples to ensure data completeness and filtering out variants with low coverage (<10× in 80% of genotypes) to improve the reliability and accuracy of the findings.

In this study, a subset of subjects examined by Wąsowska et al. [22] was utilized for the PRS calculation. It was conducted using PLINK for quality control of genotyped data and the additive model available via PRSice2 software. The PRS study was specifically tailored to the Polish population based on targeted sequencing data involving the enrichment of genes known to be associated with AMD at the time of the study [37]. Details of the genetic tests conducted on the study participants and the methods and results of PRS modeling are the subject of previous publications [22,37].

### 2.6. Fundus Images Selection

**Figure 1:**
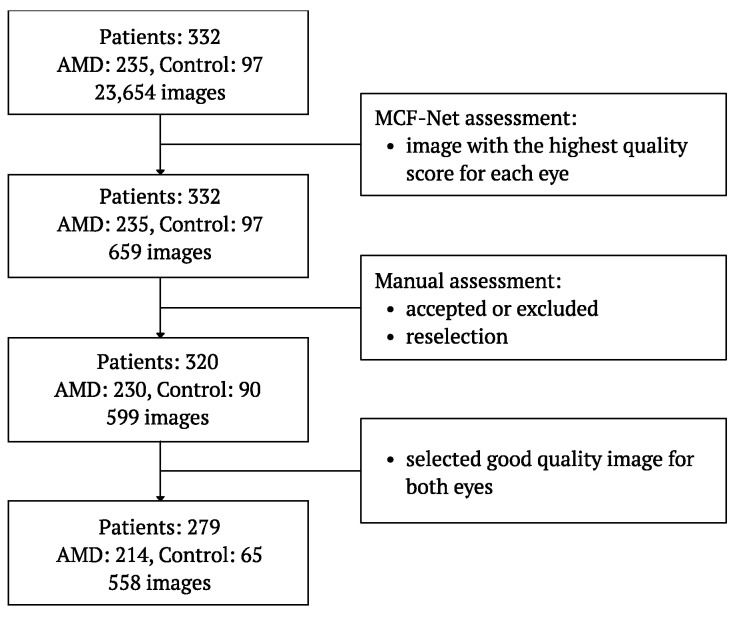
Flow chart of fundus images quality assessment and selection.

Figure 1 shows the flow chart for fundus image selection based on the automatic quality assessment tool and the manual experts’ evaluation.

The 23,654 fundus images were collected for the 332 patients who qualified for the study. For the initial image quality check, we used the pre-trained MCF-Net model [38]. The model classifies fundus images in terms of quality as good, usable, and reject, and was previously used in many studies [39,40,41,42]. Based on the results, we calculated the quality score as the sum of the predicted probabilities for the “good” and “usable” classes. We then selected the image with the highest quality score for each eye. During this process, it turned out that only one eye image was recorded for five patients in the entire dataset. The quality of 659 selected images from 332 patients was manually assessed by an expert ophthalmologist. The presence of artifacts that prevent the evaluation of the central retina, such as blur, insufficient illumination, and shadows, resulted in image rejection. In such cases, the expert manually reviewed all the images collected for the eye to choose the better quality image. Eventually, the eye was rejected if any image’s quality was insufficient. Finally, 320 patients and 599 images remained, from which we selected patients with good-quality images for both eyes. A total of 558 images were selected for further analysis: 214 from patients with AMD and 65 from subjects in the control group.

### 2.7. Deep Learning

We used the DenseNet121 deep learning model to estimate PRS from single fundus images sized 512 × 512. DenseNet121 is a deep convolutional neural network architecture commonly used in image processing for tasks like detection and classification, leveraging densely connected layers to enhance feature propagation and reduce computational costs [43]. Our preliminary experiments on a reduced dataset showed that among the various models, the complexity of the DenseNet121 architecture avoids the constant prediction problem while reducing overfitting. It is also supported by the results of previous research on using this model to extract features from fundus images for automatic classification of eye diseases, including AMD [44,45]. At the top of the network, we used a global average pooling layer and a single neuron with a linear activation function, whose output was the estimated PRS value. We used transfer learning with the initial ImageNet weights and the fine-tuning technique by unfreezing all network layers to train the model with better efficiency and reduced overfitting. The network was trained using the adaptive moment estimation (Adam) optimizer with a mean-squared error (MSE) loss function. The batch size was set to 8, and the maximum number of epochs to 50. Moreover, to prevent overfitting and achieve better model generalization, we used an augmentation of images from the training set at each epoch involving random flipping in two directions and random changes in brightness, contrast, saturation, and hue.

**Figure 2:**
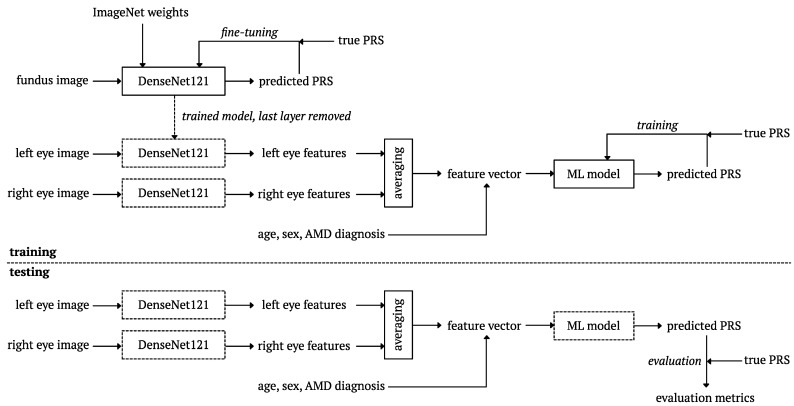
Flow chart of training and validation procedures in the hybrid model for PRS estimation based on fundus images.

After training the model, we extracted 1024 deep features from the global average pooling layer output for each eye. The feature values were fused by averaging the left and right eyes, and after completing with age, sex, and AMD diagnosis, they were used in machine learning regression models to estimate PRS for each subject. We tested this solution for the following models: Random Forest, Bayesian Ridge, AdaBoost, Extra Trees, and K Neighbors. The procedure for training and validating the proposed hybrid model is shown in Figure 2.

The validation of the proposed approach was carried out using 5-fold cross-validation with a fixed split between the test and training sets at each stage of the algorithm. When splitting, we ensured that all of one subject’s data (right and left eye images) always went into one of the sets: test or training. For the results from each model in every fold, we computed evaluation metrics to assess the correctness of the estimation of PRS. Since the proposed approach is based on a regression model, common regression evaluation metrics were used to assess its performance:

- Mean Absolute Error (MAE)
  $$MAE=\frac{1}{n}\sum_{i=1}^{n}\left|y_{i}-{\hat{y}}_{i}\right|$$
- Mean Squared Error (MSE)
  $$MSE=\frac{1}{n}\sum_{i=1}^{n}{\left(y_{i}-{\hat{y}}_{i}\right)}^{2}$$
- Root Mean Squared Error (RMSE)
  $$RMSE=\sqrt{\frac{1}{n}\sum_{i=1}^{n}{\left(y_{i}-{\hat{y}}_{i}\right)}^{2}}$$
- R^2^ (Coefficient of Determination)
  $$R^{2}=1-\frac{\sum_{i=1}^{n}{\left(y_{i}-{\hat{y}}_{i}\right)}^{2}}{\sum_{i=1}^{n}{\left(y_{i}-\bar{y}\right)}^{2}}$$
- Mean Absolute Percentage Error (MAPE)
  $$MAPE=\frac{1}{n}\sum_{i=1}^{n}\left|\frac{y_{i}-{\hat{y}}_{i}}{y_{i}}\right|\times 100$$

where:

*n* — the number of observations,
$y_{i}$ — the actual value for the *i*-th observation,
${\hat{y}}_{i}$ — the predicted value for the *i*-th observation,
$\bar{y}$ — the average of the actual values.

We also used the resulting test set PRS estimations from every fold to compute the final evaluation metrics assessing the association between deep image features extracted from fine-tuned DenseNet121 and AMD PRS.

We used the Gradient-weighted Class Activation Mapping (Grad-CAM) technique to achieve activation maps from the last convolutional layer in fine-tuned DenseNet121. Based on inputs from the test set, we generated heatmaps to examine the relationship between areas of increased model activity and morphological features of the retina that are specific to AMD.

## 3. Results

A comparison of the clinical characteristics of patients with AMD and control group participants is shown in Table 1. The result of the U Mann–Whitney test showed a significant age difference, visual acuity, and choroidal thickness between groups. The chi-square test results showed no significant difference in the sex ratio between the groups. The differences in visual acuity and choroidal thickness confirm the correct selection of study participants for both groups. The demonstrated difference in age between the groups indicates the possibility of using this variable as a model input feature to account for its potential impact on the results obtained.

The mean values and standard deviations of the results of the evaluation metrics from 5-fold cross-validation are shown in Table 2. The hybrid method combining deep features from DenseNet121 with machine learning regression methods improved results regardless of the model used. The standard deviation values indicate significant differences in results depending on the training and testing sets in each fold. This difference is particularly apparent for the DenseNet121 model and is reduced when the machine learning model is included. The best results were obtained for the Random Forest model, but the differences in MAE, MSE, and RMSE between the first four models are relatively small. The obtained value of the coefficient of determination R^2^ = 0.12 indicates a large spread of estimated values, but concerning the values of other metrics, it allows us to conclude that there is a relationship between the values of deep image features and the PRS value independently of the training set used.

**Figure 3:**
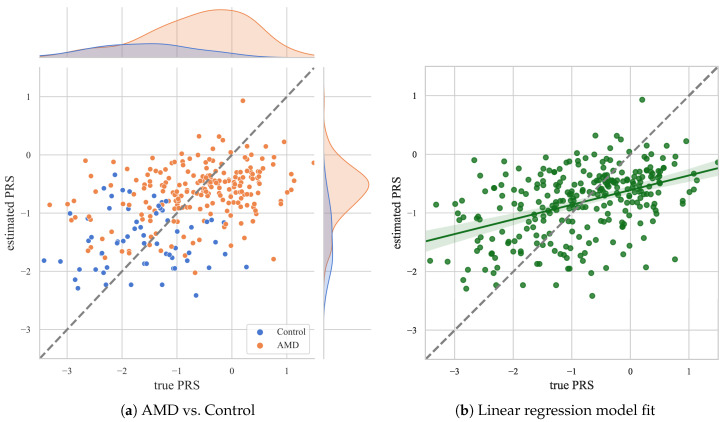
Scatter plots for the results of all folds test sets comparing true and estimated PRS values and the distributions in the control and AMD groups (**a**) and a linear regression model fit (**b**).

The final values of the metrics for all predictions from the best model from each fold are MAE = 0.74, MSE = 0.85, RMSE = 0.92, R^2^ = 0.18, MAPE = 2.41. The distribution of the resulting estimations by patients with AMD and the control group is similar to the distribution of the true PRS values (Figure 3a). The PRS values estimated by the model show an increasing trend. However, there is an overestimation in the range of low PRS values (Figure 3b), regardless of whether the data are from patients with AMD or the control group (Figure 3a).

Table 3 summarizes example fundus images combined with Grad-CAM results showing a heatmap of the convolutional neural network activation and PRS results from each analysis step.

Considering the images from the control group with healthy retinas, the algorithm highlights the optic disc as a hot spot, distinguishing it clearly from surrounding structures. If they exist, the algorithm accurately identifies and marks the areas of drusen concentrations, which leads to a higher PRS calculation than the actual PRS despite the image being classified as a control. In case number 3 of Table 3, despite the indication of several paracentral drusen, the PRS estimated by the CNN was lower than when focusing on the optic disc (cases 1 and 2 of Table 3). In fundus images depicting varying stages of AMD, the model consistently identifies crucial features indicative of disease progression. In most cases, the highlighted areas are situated prominently in the foveal and perifoveal regions. In particular, in the intermediate form of AMD, the presence of drusen clustered around the fovea is indicated, regardless of size (cases 4, 5, and 6 of Table 3). Transitioning to a late-stage AMD image in case 7 of Table 3, notable for prominent central retinal atrophy, the algorithm accurately highlights regions exhibiting severe atrophy, effectively illustrating the extent of retinal degeneration. The PRS estimated by CNN in the cases presented is much higher when a large lesion area is present and correctly indicated. These results demonstrate that the CNN model bases the decision on the prediction value on correctly learned patterns related to the occurrence of retinal lesions even though it was trained without knowledge of AMD occurrence, based only on AMD PRS values. The prediction results for lesions depend on the size of the area of abnormality and the severity of the lesions, validating the reliability of the CNN model. The absence of lesions means that the image does not consist of image features and patterns learned by the model. In this case, the model focuses on the optic disc area, returning relatively high AMD PRS prediction values ranging from −0.8 to −0.3. Increased model activity within the optic disc does not imply a link between this area and AMD PRS and may be due to stronger gradients resulting from significantly higher brightness and contrast in this area. It means that the fundus image of normal retinal morphology is challenging to the reliability of the CNN model.

The errors and artifacts observed among the obtained results depending on the quality and characteristics of the images are presented in Table 4.

In the process of selecting images where there was no better option, we chose images with small overexposures. Such artifacts appeared to have a significant impact on the resulting model performance for both control and AMD images, resulting in relatively high estimated PRS (cases 1 and 3 of Table 4). Moreover, additional artifacts, like dust on the OCT camera lens and darker areas, heighten contrast and confuse the model into misinterpreting hyperreflective lens contamination as retinal pathology (case 2 Table 4). With regard to lesions occurring within the retina, for an exemplary fundus image of a patient with an advanced neovascular (hemorrhagic) AMD, the model ignored a darker area of extravasated blood in the foveal zone, focusing on a much brighter optic disk (case 4 of Table 4).

## 4. Discussion

This study showed that using deep learning for feature extraction from fundus images, combined with a hybrid regression machine learning model, provides the ability to estimate the risk of AMD determined by polygenic analysis. The proposed convolutional neural network makes AMD PRS predictions based on abnormalities in retinal morphology. Using deep features from fundus images of both eyes in the hybrid approach significantly improved the results, indicating the potential relationship between AMD PRS and the morphological features of the retina seen on fundus images.

The understanding of the genetic basis of AMD has changed over the years. Advances in genetic research, reflected in numerous publications, have led to the development of a broader genetic panel and a multi-gene approach to the etiology and risk of AMD. For instance, as early as 2014, Lars G. Fritsche et al. discussed the polygenic risk of AMD [19]. The result of this line of thinking was GWAS, which covered a wide group of patients and allowed for a new look at the genetics of the disease in question [46]. It is worth mentioning that GWAS are continuously updated, revealing additional genetic variants associated with AMD [21,47,48]. The possibility of performing a polygenic analysis of AMD risk has opened up the search for associations between AMD PRS and complex retinal morphological factors in this disease. Previous studies in this area have shown a relationship between AMD PRS and the thickness of the outer retinal layer and the thickness of the photoreceptor layer [30,31,32]. The results of our study suggest the potential existence of other relationships seen in fundus images, not only related to changes in the thickness of individual retinal layers, particularly measured on OCT images.

Using artificial intelligence to analyze retinal images is a common practice among researchers. For years, attempts have been made to find a deep learning algorithm to aid in quickly diagnosing AMD based on the fundus images. A study conducted by Dong et al. systematically reviewed the literature on the use of artificial intelligence for the detection of AMD. The authors found that AI is a promising tool for detecting AMD, with a sensitivity of 81% and a specificity of 91% [15]. Methods for assessing the severity of changes in the central retina in patients with AMD were also analyzed [46,49]. A study performed by Bhuiyan et al. found that the algorithm could accurately stratify AMD severity, with a mean area under the receiver operating characteristic curve of 0.92. It is worth mentioning that Liu et al. found out that by combining fundus and OCT images, the number of referrals identified by ophthalmologists and AI increased significantly (scores have risen from 45 to 75 and from 53 to 85, respectively) [50]. Similar conclusions were made by Kang et al., developing a model demonstrating good performance in detecting treatment-requiring retinal diseases [51].

In addition to the automatic classification of the disease, in a study published by Ahadi et al., deep learning models were applied to predict an individual’s age from retinal fundus images, and it was shown that these predictions may be the basis for morphological and genome-wide relationship analysis [52]. In another study, Zekavat et al. demonstrated that deep learning can quantify fundus images for integration with genetic data to predict and modify disease risk [16]. In our study, we proposed a similar approach, demonstrating that deep learning is an effective tool in feature extraction from fundus images for analyzing the association of retinal morphology with AMD PRS.

From our research to date, we have achieved the greatest effectiveness in examining the relationship between PRS and changes within the OCT layers, but we treated it as a preliminary study for analyzing a larger population [33]. We made no significant progress in the search for a relationship between PRS and OCTA, where no relationship was found between the vascular network and the PRS result. This is due to the large variability of the vascular structure, the thickness of the choroid, and the interpenetration of the vascular layers of individual plexuses [53]. However, regardless of the imaging method, the multi-gene approach allows us to confirm that genetic polymorphisms have a cumulative effect on the morphology of the retina. This proves the validity of our concept that in a disease of such a polygenic nature, we should consider this aspect when looking for associations with imaging data.

Several limitations in our study must be acknowledged. One significant limitation is that this research was conducted as a single-center study, with all imaging performed on a single OCT device. It may limit the generalizability of our findings, as variations in imaging equipment could affect the performance of the AI model. Future studies should aim to replicate our findings in multi-center settings using different imaging platforms to ensure broader applicability. The study was conducted on subjects from the Polish population, which may limit the relevance of our findings to only a similar ethnic group from the European population. For other ethnic groups, the above results have only comparative significance due to the different proportions of variant frequencies and also PRS. Moreover, the model developed in this study is disease-specific, meaning it is only effective in the fundus images of patients with AMD. Other pathologies, such as diabetic retinopathy, may coexist with AMD in community or primary care clinic settings, which could confound the algorithms. The limitations of our study also include the fact that the neural network learned well to recognize light-colored retinal pathologies (drusen, scars, geographic atrophy, subretinal fibrosis), while the effectiveness of detecting lesions with a darker color than the surroundings (e.g., hemorrhagic form of AMD) was much lower. We put this down to the limited number of subjects with macular hemorrhage, which meant that the algorithm was unable to learn to recognize this type of retinal lesion. Additionally, the CNN model showed limited performance for images of a healthy retina, which means that the resulting prediction values are mainly based on learned patterns that determine abnormalities in retinal morphology. Furthermore, despite careful selection of images for quality, the model is sensitive to the presence of artifacts.

Even though the AI-based ocular disease diagnostic support systems presented in the publications have high efficiency, their use in clinical practice is still limited due to ethical issues, among other reasons [54]. Each study involving artificial intelligence as a method supporting the diagnostic process should be primarily aimed at detecting limitations that may be dangerous to health by delaying diagnosis and treatment. When implementing artificial intelligence systems for automatic disease detection, we must be aware of their basic limitations resulting from the methodology of training AI models: impaired detection of changes that rarely occur in a given disease but are unambiguous to a human who evaluates fundus images; and the situation of multimorbidity, which manifests itself in imaging by the co-occurrence of changes of a different nature, which in most models will result in the selection of only one diagnosis.

## 5. Conclusions

Complex human traits, such as AMD, result from the interaction of multiple genetic variants and environmental factors. While GWAS have identified over 100 common genetic variants associated with AMD, these variants account for only a small fraction of the disease’s heritability. This suggests that other factors, such as gene–gene interactions and epigenetic modifications, likely contribute to the development of AMD.

Identifying rare genetic variants that have a large effect on AMD risk has been challenging. However, recent advances in next-generation sequencing (NGS) have made it possible to identify these variants. NGS has also been used to identify genetic variants that are associated with the progression of AMD.

The use of AI is further enhancing our understanding of the genetic basis of AMD. AI is used to analyze large datasets of genetic and clinical data to identify patterns and associations that would be difficult to find using traditional methods. The combination of GWAS, NGS, and AI is providing new insights into the genetic basis of AMD, which is crucial for developing more effective prognostic methods and treatments for this debilitating disease.

The screening and prediction models developed in this study have the potential to be valuable public health tools for the prevention of legal blindness from AMD through telemedicine. The screening model can be used to identify individuals at risk of developing AMD, who can then be referred for further evaluation and treatment.

The models presented in our study can be utilized to verify the assignment of patients to the appropriate groups. This application is particularly useful for the analysis of large databases containing numerous images, significantly reducing the potential for human error.

The prediction model can be used in ophthalmology clinics to identify patients who require closer surveillance and better attention to modifiable risk factors and who may wish to be considered for advanced therapies.

Further validation in prospective trials will help determine the optimal utilization of these models to prevent legal blindness from AMD.

## Figures and Tables

**Table 1:** Clinical characteristics of patients with AMD and control group—mean (standard deviation).

	AMD	Control	*p*-Value
N	214	65	-
Age [years]	76.13 (7.67)	70.48 (7.28)	<0.001
Sex [male/female]	82/132	14/51	0.019
Visual acuity [logMAR]	0.65 (0.53)	0.15 (0.20)	<0.001
Choroidal thickness [µm]	229.4 (112.7)	263.5 (98.8)	<0.001

AMD—age-related macular degeneration.

**Table 2:** Evaluation metrics from different regression models for the test set, based on deep image features extracted by fine-tuned DenseNet121—mean (standard deviation).

Model	MAE	MSE	RMSE	R^2^	MAPE
Random Forest	0.75 (0.09)	0.90 (0.12)	0.95 (0.06)	0.12 (0.14)	2.45 (0.77)
Bayesian Ridge	0.78 (0.07)	0.91 (0.10)	0.95 (0.11)	0.11 (0.11)	2.47 (0.79)
AdaBoost	0.77 (0.09)	0.93 (0.14)	0.96 (0.07)	0.08 (0.05)	2.60 (0.73)
Extra Trees	0.77 (0.11)	0.95 (0.17)	0.97 (0.09)	0.06 (0.20)	2.47 (0.63)
K Neighbors	0.83 (0.11)	1.08 (0.17)	1.04 (0.08)	−0.05 (0.12)	2.52 (0.72)
DenseNet121	1.10 (0.24)	2.00 (0.74)	1.39 (0.27)	−1.00 (0.91)	3.04 (0.82)

MAE—mean absolute error; MSE—mean squared error; RMSE—root mean squared error; R^2^—coefficient of determination; MAPE—mean absolute percentage error.

**Table 3:** Selected heatmaps generated with Grad-CAM technique from last convolutional layer in DenseNet121 model.

No.	Fundus Image	Grad-CAM	Group	PRS	CNN	CNN+ML
1	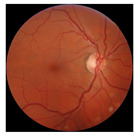	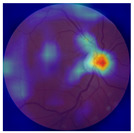	Control	−1.07	−0.57	−1.95
2	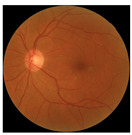	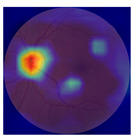	Control	−0.30	−0.85	−1.50
3	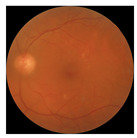	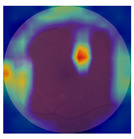	Control	−3.12	−1.53	−1.83
4	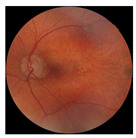	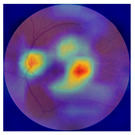	AMD	−0.99	0.29	−0.86
5	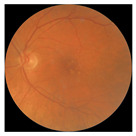	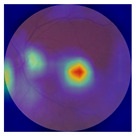	AMD	−0.23	−0.29	−0.66
6	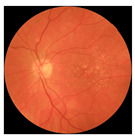	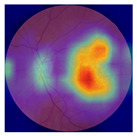	AMD	0.17	1.31	−0.75
7	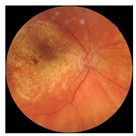	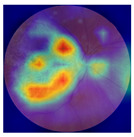	AMD	−0.43	1.16	−0.48

AMD—age-related macular degeneration; Grad-CAM—Gradient-weighted Class Activation Mapping; CNN—convolutional neural network (DenseNet121); CNN+ML—hybrid model (DenseNet121 features used in machine learning regression model).

**Table 4:** Selected artifacts and errors on heatmaps generated with Grad-CAM technique from last convolutional layer in DenseNet121 model.

No.	Fundus Image	Grad-CAM	Group	PRS	CNN	CNN+ML
1	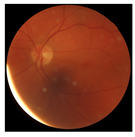	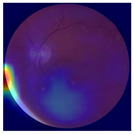	Control	−2.13	0.63	−0.34
2	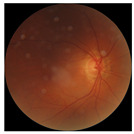	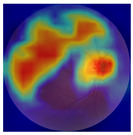	Control	−2.61	1.80	−1.48
3	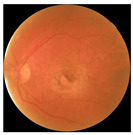	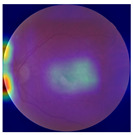	AMD	1.08	0.52	−0.60
4	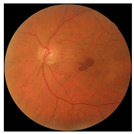	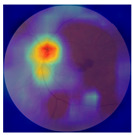	AMD	−0.39	−0.16	−0.41

AMD—age-related macular degeneration; Grad-CAM—Gradient-weighted Class Activation Mapping; CNN—convolutional neural network (DenseNet121); CNN+ML—hybrid model (DenseNet121 features used in machine learning regression model).

## Data Availability

The datasets generated and analyzed during the current study are available from the corresponding author on reasonable request.
